# Radial gradient and radial deviation radiomic features from pre-surgical CT scans are associated with survival among lung adenocarcinoma patients

**DOI:** 10.18632/oncotarget.21629

**Published:** 2017-10-06

**Authors:** Ilke Tunali, Olya Stringfield, Albert Guvenis, Hua Wang, Ying Liu, Yoganand Balagurunathan, Philippe Lambin, Robert J. Gillies, Matthew B. Schabath

**Affiliations:** ^1^ Department of Cancer Imaging and Metabolism, H. Lee Moffitt Cancer Center and Research Institute, Tampa, Florida, USA; ^2^ Department of Cancer Epidemiology, H. Lee Moffitt Cancer Center and Research Institute, Tampa, Florida, USA; ^3^ Institute of Biomedical Engineering, Bogazici University, Istanbul, Turkey; ^4^ Faculty of Biomedical Engineering, Namik Kemal University, Tekirdag, Turkey; ^5^ Department of Radiology, Tianjin Medical University Cancer Institute and Hospital, National Clinical Research Center of Cancer, Key Laboratory of Cancer Prevention and Therapy, Tianjin, PR China; ^6^ Research Institute GROW of Oncology, Maastricht University Medical Center, Maastricht, The Netherlands

**Keywords:** radiomics, radial gradient, radial deviation, lung adenocarcinoma, quantitative imaging

## Abstract

The goal of this study was to extract features from radial deviation and radial gradient maps which were derived from thoracic CT scans of patients diagnosed with lung adenocarcinoma and assess whether these features are associated with overall survival. We used two independent cohorts from different institutions for training (n= 61) and test (n= 47) and focused our analyses on features that were non-redundant and highly reproducible. To reduce the number of features and covariates into a single parsimonious model, a backward elimination approach was applied. Out of 48 features that were extracted, 31 were eliminated because they were not reproducible or were redundant. We considered 17 features for statistical analysis and identified a final model containing the two most highly informative features that were associated with lung cancer survival. One of the two features, radial deviation outside-border separation standard deviation, was replicated in a test cohort exhibiting a statistically significant association with lung cancer survival (multivariable hazard ratio = 0.40; 95% confidence interval 0.17-0.97). Additionally, we explored the biological underpinnings of these features and found radial gradient and radial deviation image features were significantly associated with semantic radiological features.

## INTRODUCTION

Lung cancer is the second most common cancer and is the leading cause of cancer-related death in the United States. Lung cancer accounts for more deaths than prostate, breast, colon, and pancreatic cancer combined [1]. Despite improvements in survival for many other cancer types over the last several decades, there has been little improvement in lung cancer patient survival, mainly because of the fact that by the time a diagnosis is made, the cancer is often in advanced stages and treatment options are limited. The five-year survival rate for all lung cancers (non-small cell lung carcinoma [NSCLC] and small cell lung cancer combined) is only 17%; and among NSCLC diagnoses, the five-year relative survival rate is 21% [2].

Pathologic staging is the most important prognostic factor for lung cancer survival [3]. However, there is marked variability in patient outcomes and survival among patients with the same stage of disease, which suggests that other factors contribute to NSCLC prognosis. These prognostic factors include sex, histology, genetic alterations in oncogenes and tumor suppressor genes, co-morbidities, and patient performance status [4–9]. Additionally, there is emerging evidence that radiological and quantitative image features are associated with patient outcomes independent of clinical covariates and patient characteristics [2, 10–14]. As diagnostic computed tomography (CT) scans are routinely obtained during the workup of lung cancer patients, image features can provide valuable and readily available complementary decision support information which could have translational implications for improved prediction of patient outcomes and further patient stratification.

With high-throughput computing, it is now possible to rapidly extract a large number of quantitative image features from standard-of-care imaging such as CT. The conversion of digital medical images into mineable high-dimensional data is a process that is known as radiomics. Radiomics is motivated by the premise that biomedical images contain information that reflects the underlying pathophysiology of the region of interest (i.e., lung tumor) and that these relationships can be revealed via conversion of images to structured data, data-mining, and statistical analysis [15]. In this study, we analyzed a set of image features extracted from radial gradient (RG) and radial deviation (RD) maps generated from thoracic CT images. For each voxel in the volume of interest (VOI), a radial deviation and a radial gradient value was calculated which in-turn formed the radial gradient and radial deviation maps. Each voxel in the radial deviation map is defined as the angle between a voxel's gradient vector and its radial vector which points towards the center of mass of the segmented lesion, whereas each voxel in the radial gradient map specified the magnitude of gradient along that voxel's radial vector. Using these maps, we generated radial gradient and radial deviation features which represent voxel-by-voxel gradient changes in the VOI. As such, we expected that these features will be sensitive to changes in tumor shape that occur along radial directions, such as lobulation and border definition, which are important predictive and prognostic features in lung cancer [11, 16, 17].

To date there have been very few studies on image features derived from radial gradient and radial deviation maps [18–20]. Features extracted from radial gradient and radial deviation maps were first used in a computer-aided detection (CAD_e_) system for eliminating false positive pulmonary nodule candidates on chest X-ray [18]. Messay et al. [19] used these features in a computer-aided diagnosis system (CAD_x_) to discriminate between benign and malignant nodules. In another study from this group, radial gradient and radial deviation image features were utilized to optimize free parameters of a CT pulmonary nodule segmentation system [20]. As such, the goal of this study was to extract features from radial deviation and radial gradient maps from pre-surgical contrast-enhanced thoracic CT scans among patients with lung adenocarcinoma and assess whether these features were associated with clinical outcomes. Additionally, we explored the potential biological underpinnings of these features by analyzing the association between radial gradient and radial deviation image features with semantic radiological features.

## RESULTS

### Patient demographics

Among the 61 patients in the training cohort, 50.8% were male, 67.2% were aged above 65 years at the date of diagnosis, and 72.1% were either stage I or II. Among the image acquisition parameters, 93.4% of the CT scans were acquired with 120 kVp, 34.4% used B41f as a convolution kernel, 65.6% had an interpolated slice thickness of 2.5 mm, and 34.4% had a pixel resolution ≥ 0.7785 (third quartile). The median time to event (overall survival) was 33.5 months for this cohort (Table 1).

**Table 1:** Patient characteristics in the training and test cohorts

Characteristic	Training cohort (N = 61)		Test cohort (N = 47)	
**Age at diagnosis, N (%)**				
< 65	20	(32.8)	25	(53.2)
≥ 65	41	(67.2)	22	(46.8)
**Sex, N (%)**				
Female	30	(49.2)	22	(46.8)
Male	31	(50.8)	25	(53.2)
**Stage, N (%)**				
I and II	44	(72.1)	32	(68.1)
III and IV	17	(27.9)	15	(31.9)
**Tumor volume, mean cm^3^ (SD)**	19.5	(29.0)	52.4	(130.0)
**Tumor max diameter, mean mm (SD)**	31.6	(13.8)	38.0	(21.5)
**Overall Survival, median months**	33.5		32.0	
**Image acquisition parameters, N (%)**				
***Voltage, kVp***				
120	57	(93.4)	40	(85.1)
130 or 140	4	(6.6)	7	(14.9)
***Convolution kernel***				
A,B	0	(0)	23	(48.9)
B30s,B60f,B70s	2	(3.3)	5	(10.7)
B30f	8	(13.1)	0	(0)
B40f	19	(31.2)	15	(31.9)
B41f	21	(34.4)	0	(0)
Other	11	(18.0)	4	(8.5)
***Interpolated slice thickness***				
1.5 mm	0	(0)	2	(4.3)
2.0 mm	8	(13.1)	13	(27.7)
2.5 mm	40	(65.6)	29	(61.7)
3.0 mm	13	(21.3)	3	(6.3)
***Pixel resolution (mm), tertiles***				
< 0.6926	20	(32.8)	6	(12.8)
≥ 0.6926 to < 0.7785	20	(32.8)	4	(8.5)
≥ 0.7785	21	(34.4)	37	(78.7)

In test cohort, there were a total of 47 patients of which 53.2% were male, 46.8% were aged above 65 years at the date of diagnosis, and 68.1% were either stage I or II. Among the image acquisition parameters, 85.1% of the CT scans were acquired with 120 kVp, 48.9% used A or B as a convolution kernel, 61.7% had an interpolated slice thickness of 2.5 mm, and 78.7% had a pixel resolution ≥ 0.7785 (third quartile). The median time to event (overall survival) was 32.0 months for this cohort (Table 1).

### Univariable analyses

After eliminating the redundant (n = 15) and non-reproducible features (n = 16), we calculated the log-rank p-values for the remaining 17 features. Out of these 17 features, two features (radial deviation outside-border separation standard deviation (SD) and radial gradient outside-border separation SD 2-dimensional (2D)) were statistically significantly associated with overall survival, (log-rank p-value ≤ 0.05) and three features (radial gradient border SD, radial gradient outside-tumor separation mean, and radial deviation tumor SD) were marginally (log-rank p-value ≤ 0.1) associated with overall survival (Table 2). The log-rank p-values for all 17 features assessed are presented in Supplementary Table 1.

**Table 2:** Log-rank tests and Cox proportional hazards model for overall survival in the training and test cohorts

Covariate	Training cohort N = 61							Test cohort N = 47			
	Log-rank P-value^1^	Univariable model^2^ OR (95% CI)	P-value	Multivariable model^3^ OR (95% CI)	P-value	Multivariable model^4^ OR (95% CI)	P-value	Multivariable model^5^ OR (95% CI)	P-value	Multivariable model^6^ OR (95% CI)	P-value
**Radial gradient border SD (feature 20)**	0.084	1.92 (0.90 - 4.11)	0.092	.	.	.	.	.	.	.	.
**Radial gradient outside-tumor separation mean (feature 35)**	0.061	0.48 (0.22 - 1.06)	0.068	**0.29 (0.12- 0.66)**	**0.003**	**0.31 (0.13 - 0.72)**	**0.006**	0.75 (0.28 - 2.03)	0.575	0.48 (0.17 - 1.37)	0.172
**Radial deviation outside-border separation SD (feature 42)**	**0.009**	**0.36 (0.16 - 0.81)**	**0.013**	**0.25 (0.11 - 0.58)**	**0.001**	**0.24 (0.10 - 0.58)**	**0.001**	**0.36 (0.16 - 0.81)**	**0.014**	**0.40 (0.17 - 0.97)**	**0.042**
**Radial gradient outside-border separation SD (2D) (feature 48)**	**0.029**	**0.43 (0.20 - 0.94)**	**0.035**	.	.	.	.	.	.	.	.
**Radial deviation tumor SD (feature 2)**	0.071	2.00 (0.92 - 4.34)	0.078	.	.	.	.	.	.	.	.
**Age**	0.439	1.38 (0.60 - 3.16)	0.444	.	.	0.83 (0.34 - 2.05)	0.690	.	.	**2.65 (1.07 – 6.60)**	**0.035**
**Sex**	0.694	1.16 (0.54 - 2.49)	0.696	.	.	1.05 (0.47 - 2.35)	0.906	.	.	1.43 (0.53 – 3.82)	0.476
**Stage**	0.085	1.95 (0.90 - 4.23)	0.093	.	.	2.14 (0.91 - 5.03)	0.082	.	.	**3.35 (1.34 – 8.36)**	**0.010**
**Tumor volume**	**0.044**	2.23 (1.00 - 4.97)	0.051	.	.	.	.	.	.	.	.

SD = standard deviation; OR = odds ratio; CI = confidence interval

Bold values are statistically significant.

^1^Log-rank p-value for each covariate for overall survival right censored at 5-years. The radiomic features were dichotomized at the median value and the clinical covariates were dichotomized based on Table 1. The univariable analyses were based on 62 patients. But, due to missing patient data (age and sex), the total sample size for the multivariable analyses was 61 patients.

^2^The independent main effect ORs for each covariate

^3^The ORs for the two image features in a single model following backward elimination that considered all features and tumor volume.

^4^The ORs for both image features identified from backward elimination adjusted for clinical covariates.

^5^The ORs for from the two image features identified in training cohort from backward elimination

^6^The ORs for both image features identified from backward elimination in training cohort adjusted for clinical covariates

**Figure 1:**
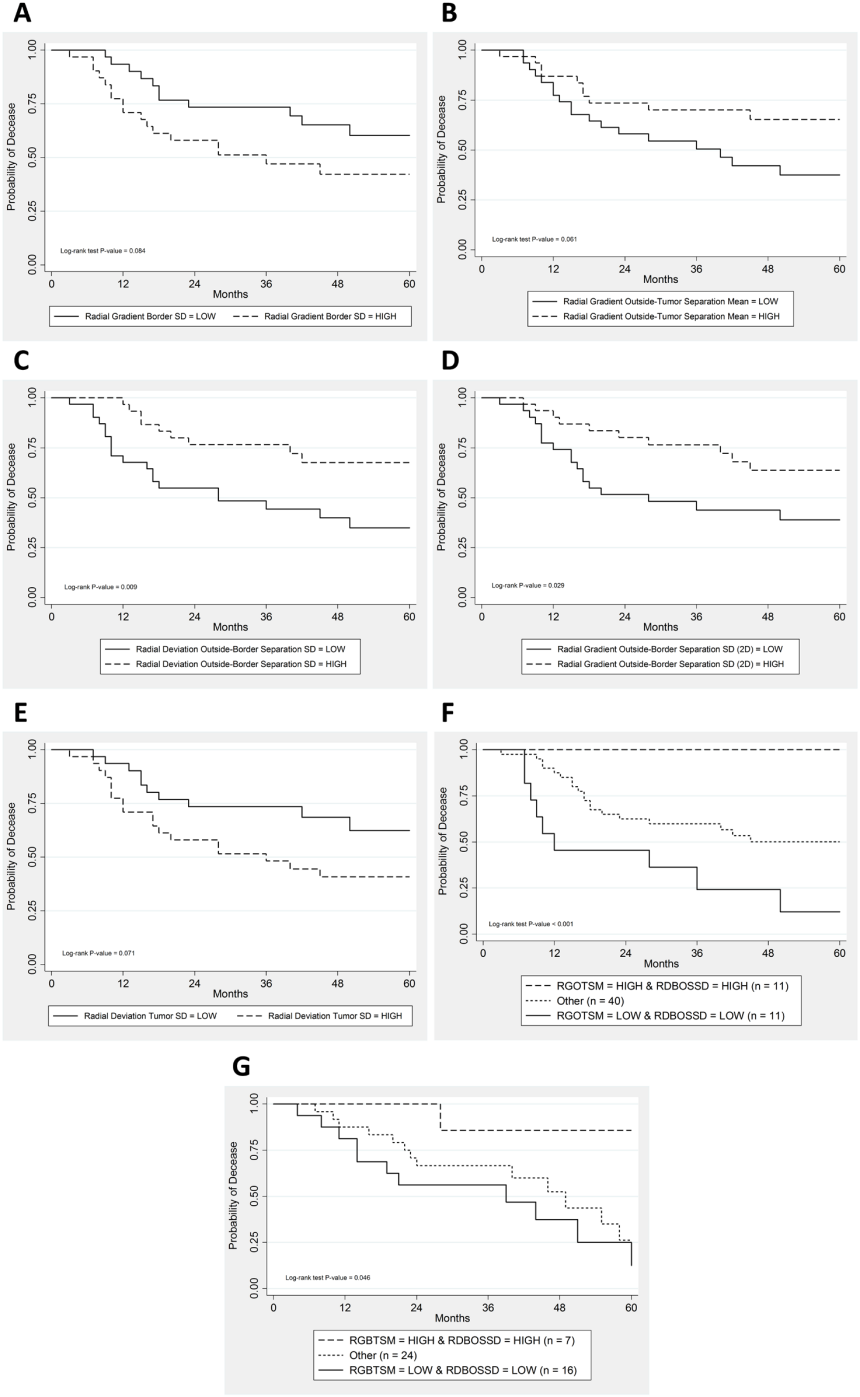
Kaplan-Meier survival curves for the following features **(A)** Radial gradient border standard deviation in the training cohort. **(B)** Radial gradient outside-tumor separation mean in the training cohort. **(C)** Radial deviation outside-border separation standard deviation in the training cohort. **(D)** Radial gradient outside-border separation standard deviation (2D) in the training cohort. **(E)** Radial deviation tumor standard deviation in the training cohort. **(F)** For the combination of radial gradient outside-tumor separation mean (RGOTSM) and radial deviation outside-border separation standard deviation (RDOBSSD) features in the training cohort. Hazard ratio with 95% confidence interval is calculated for the entire cohort (HR = 3.65; 95% CI (1.89–7.05)). **(G)** For the combination of radial gradient outside-tumor separation mean (RGOTSM) and radial deviation outside-border separation standard deviation (RDOBSSD) features in the test cohort.

The Kaplan-Meier survival curves using a median cutoff for the five features are presented in Figure 1A to 1e and the 5-year survival rates are presented in Supplementary Table 2. For the two features that were significantly associated with overall survival, tumors with high (≥ median) radial deviation outside-border separation SD (Figure 1C, Hazard Ratio [HR] = 0.36; 95% CI 0.16-0.81, p = 0.013) and radial gradient outside-border separation SD (Figure 1D, HR = 0.43; 95% CI 0.20-0.94, p = 0.035) were associated with improved overall survival (Table 2). For the three features which were marginally significant for overall survival, tumors with high radial gradient border SD (Figure 1A, HR = 1.92; 95% CI 0.90-4.11, p = 0.092) and radial deviation tumor SD (Figure 1E, HR = 2.00; 95% CI 0.92-4.34, p = 0.078) were associated with poor overall survival while high radial gradient outside-tumor separation mean was associated with improved overall survival (Figure 1B, HR = 0.48; 95% CI 0.22-1.06, p = 0.068).

In an exploratory analysis, we found low correlation between the features that were previously published in these cohorts (entropy ratio and convexity) [2] to radial deviation and gradient features in this analysis (Pearson correlation coefficient < 0.35 for all features). Hence, the radial gradient and deviation features provide orthogonal information to previously identified features.

### Multivariable analyses

To reduce the number of image features to the most meaningful subset associated with overall survival, we applied a stepwise backward elimination model using a threshold of 0.01 to identify a parsimonious model. All five features and tumor volume were considered for the inclusion in the final model. The two features that remained in a feature-only multivariable model were radial deviation outside-border separation SD (HR = 0.25; 95% CI 0.11-0.58, p = 0.001) and radial gradient outside-tumor separation mean (HR = 0.29; 95% CI 0.12-0.66, p = 0.003). To control for potential confounding, these two features were included in a multivariable Cox Model that included age, sex, and stage; and both features remained statistically significant (Table 2). We further analyzed these two features by demographics and imaging parameters and generated contingency tables. In the training cohort, none of the demographics or imaging parameters were significantly associated with radial deviation outside-border separation SD and radial gradient outside-tumor separation mean (Table 3).

**Table 3:** Demographics and imaging parameters by image features in training cohort

Covariate	Radial gradient outside-tumor separation mean			Radial deviation outside-border separation SD		
	LOW	HIGH	P- Value	LOW	HIGH	P- Value
**Sex, N (%)**						
Female	14 (45.2)	16 (53.3)	0.612	13 (43.3)	17 (54.8)	0.446
Male	17 (54.8)	14 (46.7)		17 (56.7)	14 (45.2)	
**Age, N (%)**						
< 65	8 (25.8)	12 (40.0)	0.283	8 (26.7)	12 (38.7)	0.416
≥ 65	23 (74.2)	18 (60.0)		22 (73.3)	19 (61.3)	
**Stage, N (%)**						
I/II	21 (67.8)	23 (77.4)	0.570	23 (77.4)	21 (67.7)	0.570
III/IV	10 (32.2)	7 (22.6)		7 (22.6)	10 (32.3)	
**5- year survival, %**	37.4%	65.3%	0.061	34.9%	67.7%	**0.009**
**Voltage, kVp, N (%)**						
120	28 (90.3)	29 (96.7)	0.612	26 (86.7)	31 (100.0)	0.053
130 or 140	3 (9.7)	1 (3.3)		4 (13.3)	0 (0)	
**Convolution kernel, N (%)**						
A,B	0 (0)	0 (0)	0.270	0 (0)	0 (0)	0.700
B30s,B60f,B70s	2 (6.7)	0(0)		1(3.2)	1(3.3)	
B30f	6 (20.0)	2 (6.4)		3 (9.7)	5 (16.7)	
B40f	7 (23.3)	12 (38.7)		12 (38.7)	7 (23.3)	
B41f	10 (33.3)	11 (35.5)		9 (29.0)	12 (40.0)	
Other	5 (16.7)	6 (19.4)		6 (19.4)	5 (16.7)	
**Interpolated slice thickness, N (%)**						
1.5 mm	0 (0)	0 (0)	0.189	0 (0)	0 (0)	0.861
2.0 mm	5 (16.1)	3 (10.0)		3 (10.0)	5 (16.1)	
2.5 mm	17 (54.8)	23 (76.7)		20 (66.7)	20 (64.5)	
3.0 mm	9 (29.1)	4 (13.3)		7 (23.3)	6 (19.4)	
**Pixel resolution, tertiles N (%)**						
< 0.6926 mm	7 (22.6)	13 (43.3)	0.146	7 (23.3)	13 (41.9)	0.172
≥ 0.6926 and < 0.7785 mm	10 (32.3)	10 (33.3)		13 (43.3)	7 (22.6)	
> 0.7785 mm	14 (45.1)	7 (23.4)		10 (33.4)	11 (35.5)	

^1^Numbers inside parenthesis are the percentage values.

To determine if these findings could be replicated in an external patient cohort, we analyzed these two features in a test cohort (Table 2) using the median threshold values obtained from the training cohort and found that radial deviation outside-border separation SD was statistically significant (Supplementary Figure 1B, HR = 0.36; 95% CI 0.16-0.81, p = 0.014) but radial gradient outside-tumor separation mean was not found to be statistically significant (Supplementary Figure 1A, HR = 0.75; 95% CI 0.28-2.03 p = 0.575) (Table 2). However, for both features, the point estimates were inversely associated with risk of death. When these two features were included in a multivariable Cox model that included age, sex and stage, radial deviation outside-border separation SD was statistically significant (HR = 0.40; 95% CI 0.17-0.97, p = 0.042) along with age (HR = 2.65; 95% CI 1.07-6.60, p = 0.035) and stage (HR = 3.35; 95% CI 1.34-8.36, p = 0.010) (Table 2). Additionally, among early stage lung cancer patients (stage I and II), we found that radial deviation outside-border separation SD was statistically significantly associated with survival in the training cohort (log-rank p-value = 0.031) and marginally significant in the test cohort (log-rank p-value = 0.097). None of the patient demographics or imaging parameters were significantly associated with radial gradient outside-tumor separation mean and radial deviation outside-border separation SD except pixel resolution was found to be significantly associated with radial gradient outside-tumor separation mean in test cohort (p = 0.010, Supplementary Table 3).

### Combinatorial analyses

In exploratory analyses, we assessed the combinatorial effects of radial deviation outside-border separation SD and radial gradient outside-tumor separation in the training and test cohorts (Figure 1F and 1G, respectively and Supplementary Table 2). In both cohorts we found that patients who had high values (> median) for both features had statistically significantly better survival compared to patients who had low values (≤ median) for both features. We also explored the subset of early stage patients (stage I and II) and found that the combinatorial effect was also statistically significant for overall survival in the training cohort (Log-rank P-value = 0.020). Although the survival pattern was similar in the test cohort, it did not reach statistical significance (Log-rank P-value = 0.19).

### Associations with semantic radiological features

We found three RD/RG radiomic features that were statistically significantly associated with three semantic features (Table 4): lobulation, pleural attachment, and border definition. Importantly, the replicated feature was significantly associated with border definition. Specifically, cancers with a well-defined border were significantly more likely to have high (> median) radial deviation outside-border separation SD. These analyses were restricted to the training cohort only.

**Table 4:** Association between semantic features and radial gradient and radial deviation features

Feature No.	Feature name	*Lobulation*			
		Absent	Present		P- Value
**48**	**Radial gradient outside-border separation SD (2D), N (%)**				
	LOW	27 (60.0)	4 (23.5)		**0.021**
	HIGH	18 (40.0)	13 (76.5)		
		***Pleural attachment***			
		**Absent**	**Present**		
**20**	**Radial gradient border SD, N (%)**				
	LOW	28 (62.2)	3 (17.7)		**0.004**
	HIGH	17 (37.8)	14 (82.3)		
		***Border definition***			
		**Well defined**	**Poorly defined**	**Other^1^**	
**20**	**Radial gradient border SD, N (%)**				
	LOW	13 (81.3)	8 (42.1)	10 (37.0)	**0.015**
	HIGH	3 (18.7)	11 (57.9)	17 (63.0)	
**42**	**Radial deviation outside-border separation SD^2^, N (%)**				
	LOW	4 (25.0)	9 (47.4)	18 (66.7)	**0.029**
	HIGH	12 (75.0)	10 (52.6)	9 (33.3)	
**48**	**Radial gradient outside-border separation SD (2D), N (%)**				
	LOW	4 (25.0)	14 (73.7)	13 (48.2)	**0.018**
	HIGH	12 (75.0)	5 (26.3)	14 (51.8)	

^1^Tumor with neither a well or poorly-defined border.

^2^This feature was replicated and found to be statistically significantly associated with survival in both the training cohort and test cohort.

## DISCUSSION

In this study we extracted radial gradient and radial deviation image features to determine whether they are associated with lung cancer patient survival. Of the 48 features that were extracted, 31 features were eliminated because they were not reproducible or they were redundant. The remaining 17 features were subjected to statistical analysis resulting in a parsimonious model containing two highly informative features associated with lung cancer survival. One of the two features (radial deviation outside-border separation SD) was replicated and found to be statistically significantly associated with overall survival in a separate external cohort (test cohort) of lung cancer patients.

**Figure 2:**
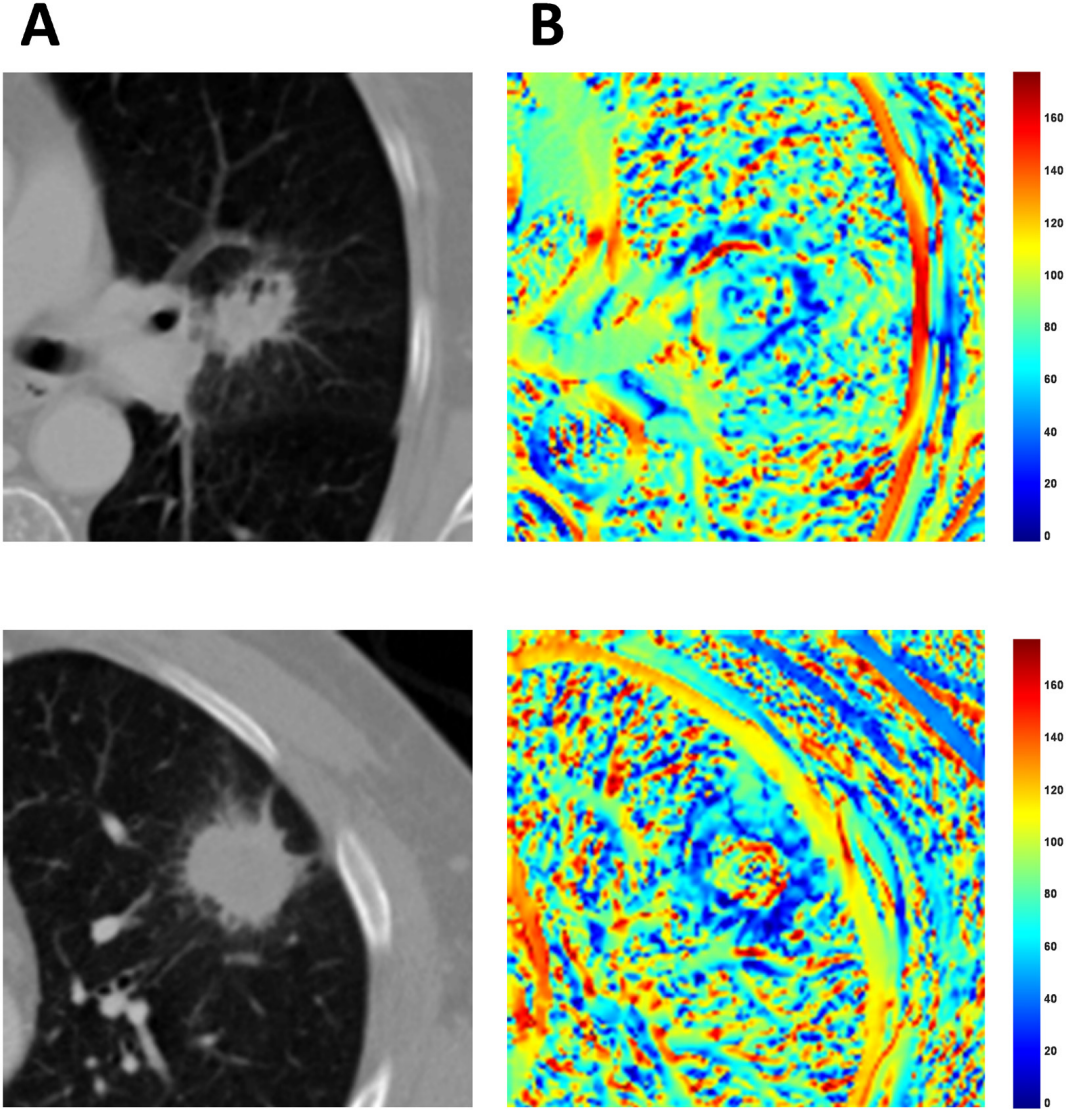
Volume of interests (VOI) for two lung cancer patients with extreme differences in clinical outcomes Radial deviation image features for the corresponding VOIs for these lung cancer patients. The top row **(A)** is a tumor of a patient (Patient ID [PID]: 33) who deceased after 9 months and the second row **(B)** is a patient (PID: 75) with who had an ongoing survival after 60 months.

Radiomics is motivated by the premise that quantitative image features reflect the underlying pathophysiology of tumors. In Figure 2A and 2B we present the volume of interest (VOI) and corresponding radial deviation maps for two patients with substantially different clinical outcomes. The patient (Figure 2A) with short survival was deceased after 9 months and had a low (< median) radial deviation outside-border separation SD value while the second patient (Figure 2B) was still alive after 60 months had a high (>median) radial deviation outside-border separation SD value. In the original CT-image, both patients have similarly-sized tumors that are speculated; however, the VOI for each radial deviation image have considerably different heat map appearances. By quantifying and analyzing these differences, as performed in this study, we have shown that RD/RG features may have clinical utility by differentiating patients with an aggressive disease and poor patient outcomes versus patients with more indolent disease and improved outcomes. Additionally, by analyzing the correlations of RD/RG features with semantic radiological features, we may have revealed their potential biological underpinnings. Specifically, we found three radial gradient and radial deviation features that were significantly associated with tumor lobulation, pleural attachment, and border definition (Table 4). The replicated feature, which was associated with lung cancer survival in both cohorts, was statistically significantly associated with border definition which has been previously reported to be a prognostic factor in lung cancer [11]. In the current analysis, patients who had well defined border definition were significantly associated with high radial deviation outside-border separation SD. As such, these analyses suggest that radial gradient and radial deviation features may be capturing clinically and biologically relevant radiological information of lung cancer tumors.

Radial image features have been previously applied in chest CT CAD systems to discriminate benign and malignant nodules, and optimize free parameters of tumor segmentation [19, 20]. However, in these previous studies, [19, 20] means and standard deviations were calculated from two different masks (region inside tumor and region outside tumor) on the radial gradient and radial deviation maps. By contrast, we calculated means and standard deviations from four different masks (tumor mask, border mask, core mask, and outside mask). To the best of our knowledge, the current study is the first to analyze RD/RG features for their association with lung cancer survival and their association with radiological semantic features.

In this study, we extracted and analyzed unique and new features from available training and test cohorts originally published by Grove et al. [2]. In the previous study, Grove et al. [2] reported that convexity and entropy ratio features were significantly associated with overall survival in the training cohort. Thus, we analyzed a model that included the convexity and entropy ratio features, RD/RG features, and patient characteristics and found that radial deviation outside-border separation SD (HR = 0.21), radial gradient outside-tumor separation mean (HR = 0.21), and entropy ratio (HR = 3.28) were statistically significantly associated with overall survival in the training cohort. However, when the remaining three features were analyzed in the test cohort, only radial deviation outside-border separation SD (HR = 0.34) was found to be statistically significant.

Quantitative image features have the potential to complement and improve current precision medicine. Limitations of tumor-based biomarkers are: they can be subjective to sampling bias due to the heterogeneous nature of tumors, the requirement of tumor specimens for biomarker testing, where the assays can be timely and expensive [2, 15, 21]. In contrast, radiomic features can be extracted in real-time from standard-of-care images, do not require timely and often expensive laboratory testing, are not subject to sampling bias and artifact, and are non-invasive. Importantly, radiomic analyses do not subject patients to additional radiation exposure since standard-of-care images are utilized, and radiomic features represent the phenotype of the entire tumor in 3D and not just the portion that was subjected to biomarker testing. Indeed, there is precedence that quantitative image features provide valuable and potentially translational information in lung cancer patient outcomes. Previous studies have shown that tumor shape and density are related to lung cancer survival [2, 13, 21, 22]. Additionally, as tumor shape becomes more eccentric, it has a higher probability of metastatic disease [2], and solid lesions that are differentiable from their outside environment and have high contrast edges and tend to be less aggressive [21]. Furthermore, tumors that are connected to lung wall are also associated with poor prognosis [21, 23].

We acknowledge some limitations and strengths to this study. First, we utilized an available set [2] of modestly sample sized training and test cohorts. However, we extracted and analyzed features that are unique from the prior work [2] and, importantly found an image feature to be significant in both the training and test cohorts. We applied a rigorous feature reduction approach to eliminate correlated and non-reproducible features, and we utilized a backward reduction approach to identify a single parsimonious model containing the most important features. We acknowledge in the combinatorial analyses that there were limited numbers of patients in the subgroups and we do not have disease-free survival data for these cohorts. A potential limitation of radiomic studies is the range of image acquisition parameters and modalities used [24] which can make it difficult to standardize image features and limit the robustness of computer-extracted features. In future studies, we will investigate interpolation methods to harmonize all data to a smaller range of slice thickness and pitch, which we hypothesize, will reduce some acquisition associated variability. However, we found that the image acquisition parameters were not associated with radial deviation outside-border separation SD for either cohort (Table 3 and Supplementary Table 3). Despite the fact that the study was limited to patients with lung adenocarcinoma, we removed potential histological differences in our analyses and we believe that this study had numerous strengths that outweigh the potential limitations.

In conclusion, this study identified a radial gradient and radial deviation image feature that was statistically significantly associated with lung cancer survival in both training and test cohorts even after adjusting for clinical covariates. Our analyses also revealed a novel combinatorial association of two features which differentiates patients with aggressive disease versus patients with indolent disease, and this was replicated in the test cohort. As such, these findings may have clinical utility to sub-stratify patients based on clinical outcome and identify patients that may need more aggressive treatment such as neo-adjuvant chemotherapy and aggressive follow-up and management. These features will require confirmation in additional studies and lung cancer patient cohorts.

## MATERIALS AND METHODS

### Lung cancer patients

This retrospective study was approved by the Institutional Review Boards at the University of South Florida and Maastricht University Medical Centre. There were two separate cohorts used in this study that have been described elsewhere [2]. Briefly, the training cohort included 61 patients from the H. Lee Moffitt Cancer Center (MCC) & Research Institute, Tampa, Florida and the test cohort included 47 patients from the Maastricht Radiation Oncology Clinic (MAASTRO), Maastricht, Netherlands. All patients were diagnosed with lung adenocarcinoma and underwent surgical resection as first course of therapy. Pre-treatment contrast enhanced CT scans were acquired within two months prior to surgery. Both cohorts included diagnostic pre-treatment contrast-enhanced CT scans acquired between 2006 and 2009 and clinical data including demographics, histology, stage, and vital status information. Follow-up for vital status information occurs annually through passive and active methods.

### Patient data

For the training cohort, clinical data were obtained from Moffitt's Cancer Registry, which abstracts self-reported patient data and clinical information from patient medical records. Follow-up information for vital status occurs annually through passive and active methods. For this analysis, vital status was updated for the Moffitt patients since the previously published report [2]. Pathologic TNM staging was utilized when available and clinical stage was used if pathologic staging was unknown. Smoking status was categorized as ever smoker (current or former smoker) or never smoker. Similar data were abstracted and databased from MAASTRO for the test cohort patients.

### Tumor segmentation

All tumors were segmented using an in-house single-click ensemble segmentation algorithm on the Lung Tumor Analysis (LuTA) software program platform (Definiens Developer XD©, Munich, Germany) [25]. After applying the single click approach, the tumor delineations were inspected and edited if needed by a resident expert radiologist. The lung and tumor mask images obtained from LuTA software program were then imported into MATLAB® (Mathworks, Natick, MA) for image feature extraction as described below.

### Radial gradient and radial deviation maps and features

Development of the radial gradient and radial deviation image features has been previously described [18, 19]. In our study, after the tumors were segmented and center of mass of the tumor was automatically detected, 48 features were extracted from the radial gradient and radial deviation maps (Supplementary Table 4) bounded by different masks (described below) which were derived from tumor delineation masks using morphological operations. Since there were variations in image acquisition parameters, we performed tri-linear interpolation by a factor of two for scans acquired with a slice thickness of ≥ 4 mm on the z-axis to create homogeneous spacing between scans. Additionally, pixels were interpolated tri-linearly in x and y directions to 2.50 mm x 2.50 mm.

**Figure 3:**
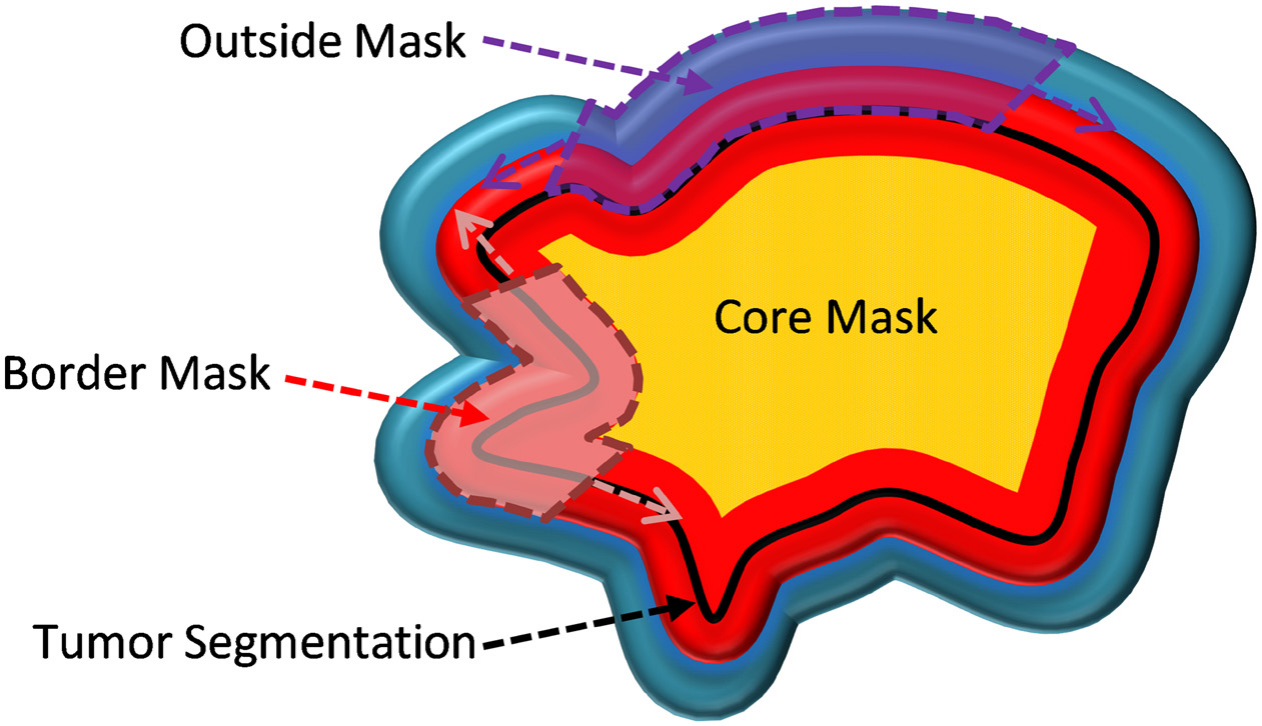
Cartoon image of the four tumor masks The region inside the black line is the tumor mask, the yellow area is the core mask, the red area is the border mask, and the region outside the black line is the outside mask (combination of the half part of red region and whole blue region).

The masks used were ‘tumor mask’, ‘border mask’, ‘core mask’, and ‘outside mask’. The tumor mask was the region that was delineated semi-automatically using Definiens Developer XD© software (Definiens, Inc., Cambridge, MA). The border mask is a “doughnut-shaped” region that was created by subtracting the two masks which are formed by a dilation operation followed by an erosion operation on the tumor mask. The region obtained after the erosion operation is the core mask. Structural elements radii used for dilation and erosion morphological operations were 7.5 mm and 12.5 mm for smaller tumors (major axis length (2D) <100 mm) and 10.0 mm and 15.0 mm for larger sized tumors (major axis length (2D) ≥ 100 mm). The outside mask was created by implementing dilation to the tumor mask followed by the subtraction of the tumor mask from the dilated region. The structural element used for the dilation morphological operation was 17.5 mm pixels for smaller tumors and 22.5 mm for larger sized tumors (Figure 3). All masks were additionally bounded to the lung parenchyma mask so that the VOIs did not exceed outside of the lung region.

The features were created using the four masks for both 2-dimensional and 3-dimensional. All 2-dimensional (2D) features were computed on the slice which included the center of mass of the segmentations. The 3-dimensional features were a natural extension of the 2D features and were calculated after the first and the last slices of the tumor segmentations were removed in order to reduce the partial volume effects. The separation features were calculated as the difference divided by the sum inside the analyzed masks (e.g., the radial deviation mean outside-border separation feature is the difference of outside radial deviation mean and the border radial deviation mean divided by the sum of the two).

**Figure 4:**
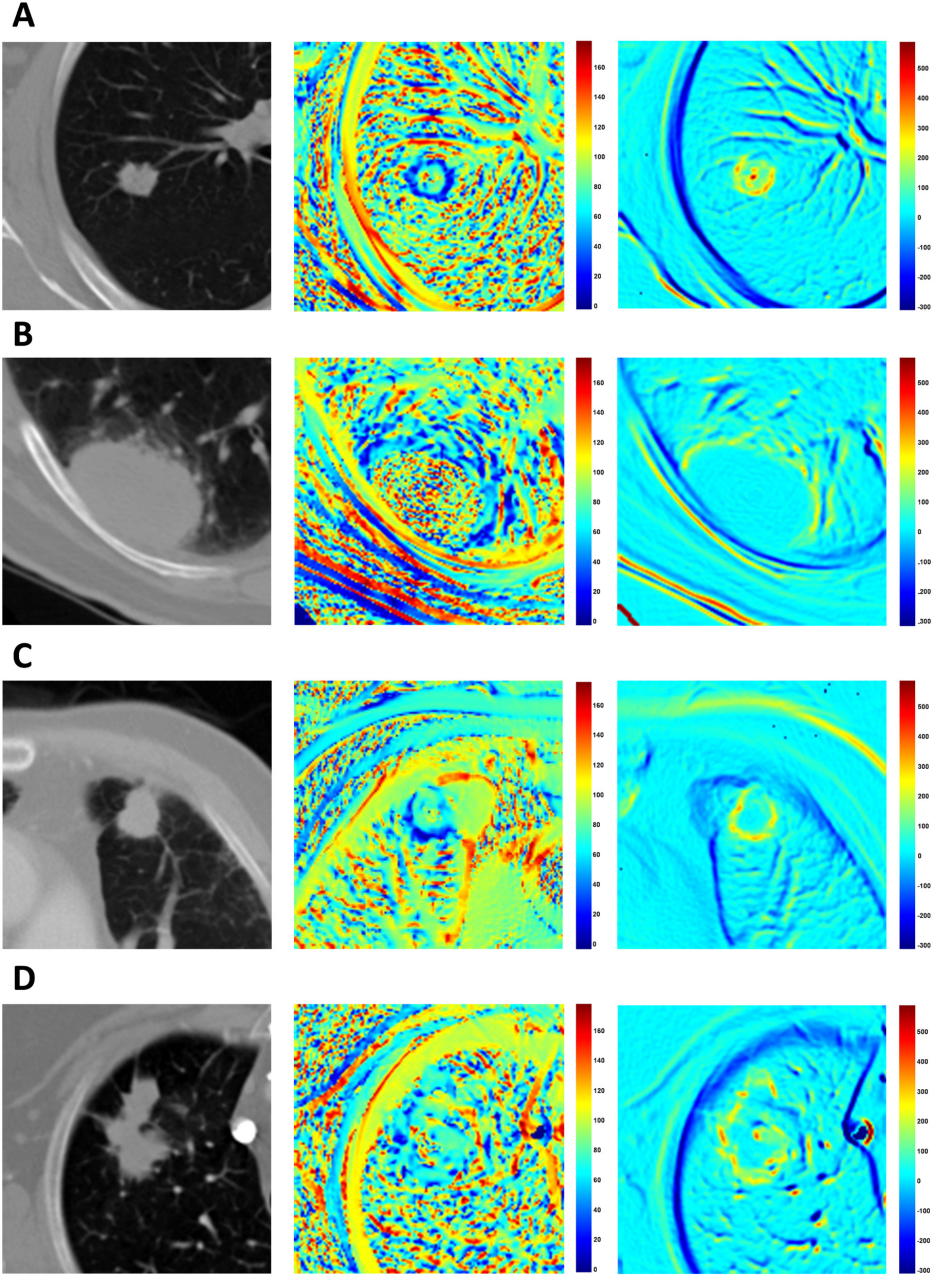
Examples of radial deviation (middle column) and radial gradient (right column) maps **(A)** An example of a tumor which yields high contrast to the lung field and a round shape and hence has lower radial deviation angles pointing to the center of mass (PID: 150). **(B)** The standard deviation of radial deviation around border and outside regions are both high, making the separation value between them small (PID: 144). **(C)** The tumor shown has a round shape and has low radial deviation angle on border regions near the lung parenchyma but has heterogeneous values on the border to the lung wall (PID: 108). **(D)** Example tumor with an irregular shape making the radial deviation and radial gradient values heterogeneous (PID: 69).

Example radial gradient and radial deviation maps for the slices that contain the center of mass of tumors are presented in Figure 4. The radial deviation values along the border lines are close to 0° when the tumors have a more spherical shape (both vectors point the same direction) and they are higher when the tumor is irregularly shaped (Figure 4A and 4D). The radial gradient values are also affected by shape along with the gradient contrast that the tumors have in respect to their microenvironment (outside region). As a result, round shaped lesions tended to have lower radial deviation values while irregularly-shaped lesions have higher radial deviation values (Figure 3A and 3D). When the lesions are attached to the lung wall, despite the fact that tumor shape along the border is restricted to a rounder shape, the low gradient on the intersection of the wall to the tumor creates deviating radial deviation values and diverges (Figure 4B and 4C). The magnitude of the gradient along the lung wall is close to zero (no gradient), as such radial gradient values are also lower along the lung wall. Radial gradient along the border is also affected by the shape of tumor, but it is furthermore influenced by how solid the tumor is in respect to its outside microenvironment. Solid tumors favor higher gradient values on border regions while semi-solid tumors have lower values (Figure 4A and 4D).

### Elimination of redundant and non-reproducible features

To eliminate the non-reproducible features we used the Reference Image Database to Evaluate Therapy Response (RIDER) dataset and calculated concordance correlation coefficient (CCC) between test-re-test scans. The RIDER dataset is a National Cancer Center (NCI) sponsored project for the guidance of integrating quantitative features across different institutions. The dataset is publicly available in National Biomedical Imaging Archive [26]. A total of 32 patients with unenhanced test-retest chest CT scans were acquired within 15 minutes of each other. The CCCs were calculated to quantify the reproducibility between consecutive scans for patients. Theoretically, CCC values range from -1 to 1, where 1 indicates a perfect correlation between two variables.

We calculated the CCCs for the 48 radial features and eliminated features that had a CCC < 0.80. As a result, 15 were features were dropped and the remaining 33 features were assessed for correlation using Pearson's correlation coefficient. When two or more features resulted in a Pearson's correlation coefficient greater than 0.80, we eliminated the feature(s) with the higher absolute column-wise correlation mean. Subsequently, 16 features were eliminated and the remaining 17 features enumerated in Supplementary Table 4 were subjected to statistical analysis.

### Radiological semantic features

Radiological semantic features for the training cohort were extracted by a clinical radiologist (YL with more than 7 years of experience) who was blinded to survival status and RD/RG status of the patients. Supplementary Table 5 contains the 13 radiological semantic features that were extracted from the CT scans of the subjects. Briefly, in terms of morphologic characteristics, the presence or absence of fissure attachment (defined as a tumor that attaches to the fissure; tumor's margin is obscured by the margin), pleural attachment (defined as tumor attaches to the pleura other than fissure; tumor's margin is obscured by the pleura), lobulation, concavity, air bronchogram [27], calcification, attachment to vessel, and pleural retraction were assessed. We also evaluated the following features, which have been defined elsewhere: dominant attenuation pattern [27], shape, border definition, spiculation [28, 29], bubble-like lucency [28], cavitation [30].

### Statistical analyses

All statistical analyses were performed using Stata/MP 14.2 (StataCorp LP, College Station, TX). All image features were dichotomized at their median value. Differences in image features by demographic features and semantic features were tested using Fisher's exact test for categorical variables and Student's t-test for continuous variables. Survival analyses were performed using Cox Proportional Hazard Regression, Kaplan-Meier curves, and log-rank tests. All survival data were right-censored at 5 years. To reduce the number of features and covariates to a single parsimonious model, a backward elimination approach was applied. The features that were identified as the most informative in the training cohort were then tested in the test cohort.

## SUPPLEMENTARY MATERIALS FIGURES AND TABLES



## Abbreviations

RD: Radial Deviation
RG: Radial Gradient
SD: Standard Deviation
NSCLC: Non-small Cell Lung Cancer
CT: Computed Tomography
VOI: Volume of Interest
CAD_e_: Computer-aided Detection
CAD_x_: Computer-aided Diagnosis
HR: Hazard Ratio
CI: Confidence Interval
MCC: H. Lee Moffitt Cancer Center
MAASTRO: Maastricht Radiation Oncology Clinic
LuTA: Lung Tumor Analysis Software
2D: 2-Dimensional
3D: 3-Dimensional
CCC: Concordance Correlation Coefficient
RIDER: Reference Image Database to Evaluate Therapy Response
NCI: National Cancer Center
