# Supplementary material for: Radial gradient and radial deviation radiomic features from pre-surgical CT scans are associated with survival among lung adenocarcinoma patients
Source: Oncotarget. 2017 Oct 6;8(56):96013–26. doi: 10.18632/oncotarget.21629 (PMC5707077; doi:10.18632/oncotarget.21629)
Supplement: Supplementary file 1 [file oncotarget-08-96013-s001.pdf]

Radial gradient and radial deviation radiomic features from pre-surgical CT scans are associated with survival among lung adenocarcinoma patients

SUPPLEMENTARY MATERIALS

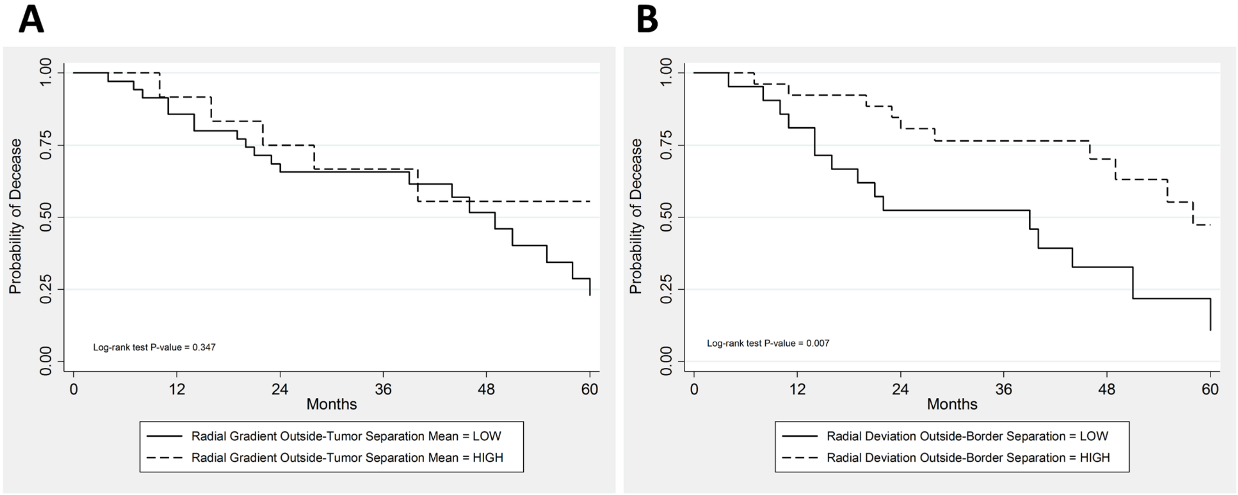

Supplementary Figure 1: Kaplan-Meier survival curves for the features in the test cohort. **(A)** Radial gradient outside-tumor separation. **(B)** Radial deviation outside-border separation standard deviation.

Supplementary Table 1: Log-rank p-values for the 17 features those are reproducible and non-redundant

No.	Features	Log-rank P-value
2	Radial deviation tumor SD	0.0706
5	Radial deviation tumor mean (2D)	0.7045
7	Radial gradient tumor mean (2D)	0.6970
8	Radial gradient tumor SD (2D)	0.6728
12	Radial gradient core SD	0.9490
20	Radial gradient border SD	0.0838
21	Radial deviation border mean (2D)	0.8747
29	Radial deviation outside mean (2D)	0.9802
30	Radial deviation outside SD (2D)	0.1111
32	Radial gradient outside SD (2D)	0.8253
33	Radial deviation outside-tumor separation mean	0.3217
34	Radial deviation outside-tumor separation SD	0.6462
35	Radial gradient outside-tumor separation mean	0.0608
36	Radial gradient outside-tumor separation SD	0.7110
41	Radial deviation outside-border separation mean	0.3217
42	Radial deviation outside-border separation SD	**0.0090**
48	Radial gradient outside-border separation SD (2D)	**0.0292**

Supplementary Table 2: 5-year survival rates for the image features[1]

	5-year survival rate	Log-rank P-value
**Five features in the training cohort**		
**Figure 1a. Radial gradient border standard deviation**		0.084
High	42.2%	
Low	60.2%	
**Figure 1b. Radial gradient outside-tumor separation mean**		0.061
High	65.3%	
Low	37.4%	
**Figure 1c. Radial deviation outside-border separation SD**		**0.009**
High	67.7%	
Low	34.9%	
**Figure 1d. Radial gradient outside-border separation SD (2D)**		**0.029**
High	63.8%	
Low	38.9%	
**Figure 1e. Radial deviation tumor SD**		0.071
High	40.8%	
Low	62.4%	
**Combinatorial Features**		
**Figure 1f. Training cohort**		**<0.001**
High/High	100.0%	
High/Low or Low/High	50.2%	
Low/Low	12.1%	
**Figure 1g. Test cohort**		**0.046**
High/High	85.7%	
High/Low or Low/High	26.3%	
Low/Low	12.5%	

[1]Image features were dichotomized at the median value so that "High" ≥ median value and "Low" < median value.

Supplementary Table 3: Demographics and imaging parameters by image features in the test cohort

	Radial gradient outside-tumor separation mean			Radial deviation outside-border separation SD		
**Covariate**	**Low**	**High**	**P- Value**	**Low**	**High**	**P- Value**
**Sex, N (%)**						
Female	17 (48.6)	5 (41.7)	0.747	8 (38.1)	14 (53.9)	0.381
Male	18 (51.4)	7 (58.3)		13 (61.9)	12 (46.1)	
**Age, N (%)**						
< 65	21 (60.0)	4 (33.3)	0.180	11 (52.4)	14 (53.9)	1.000
≥ 65	14 (40.0)	8 (66.7)		10 (47.6)	12 (46.1)	
**Stage, N (%)**						
I/II	25 (71.4)	7 (58.3)	0.481	11 (52.4)	21 (80.8)	0.059
III/IV	10 (28.6)	5 (41.7)		10 (47.6)	5 (19.2)	
**5- year survival, %**	23.0%	55.6%	0.347	10.9%	47.4%	**0.007**
**Voltage, KVp, N (%)**						
120	29 (82.9)	11 (91.7)	0.659	17 (81.0)	23 (88.5)	0.684
130 or 140	6 (17.1)	1 (8.3)		4 (19.0)	3 (11.5)	
**Convolution kernel, N (%)**						
A, B	18 (51.4)	5 (41.7)	0.905	9 (42.9)	14 (53.8)	0.877
B30s,B60f,B70s	4 (11.4)	1 (8.3)		3 (14.3)	2 (7.7)	
B30f	0 (0.0)	0(0.0)		0 (0.0)	0 (0.0)	
B40f	10 (28.6)	5 (41.7)		7 (33.3)	8 (30.8)	
B41f	0 (0.0)	0(0.0)		0(0.0)	0(0.0)	
Other	3 (8.6)	1 (8.3)		2 (9.5)	2 (7.7)	
**Interpolated slice thickness, N (%)**						
1.5 mm	2 (5.7)	0 (0.0)	0.219	2 (9.5)	0 (0.0)	0.381
2.0 mm	12 (34.3)	1 (8.3)		7 (33.3)	6 (23.1)	
2.5 mm	19 (54.3)	10 (83.4)		11 (52.4)	18 (69.2)	
3.0 mm	2 (5.7)	1 (8.3)		1 (4.8)	2 (7.7)	
**Pixel resolution, tertiles N (%)**						
< 0.6926	3 (8.6)	3 (25.0)	**0.010**	2 (9.5)	4 (15.4)	0.877
≥ 0.6926 and < 0.7785	1 (2.9)	3 (25.0)		2 (9.5)	2 (7.7)	
≥ 0.7785	31 (88.5)	6 (50.0)		17 (81.0)	20 (76.9)	

Numbers inside parenthesis are the percentage values.

Supplementary Table 4: Radial gradient and radial deviation imaging features

No.		Features
1	C	Radial deviation tumor mean
**2**	**F**	**Radial deviation tumor SD**
3	C	Radial gradient tumor mean
4	C	Radial gradient tumor SD
**5**	**F**	**Radial deviation tumor mean (2D)**
6	R	Radial deviation tumor SD (2D)
**7**	**F**	**Radial gradient tumor mean (2D)**
**8**	**F**	**Radial gradient tumor SD (2D)**
9	R	Radial deviation core mean
10	R	Radial deviation core SD
11	R	Radial gradient core mean
**12**	**F**	**Radial gradient core SD**
13	R	Radial deviation core mean (2D)
14	R	Radial deviation core SD (2D)
15	R	Radial gradient core mean (2D)
16	R	Radial gradient core SD (2D)
17	C	Radial deviation border mean
18	C	Radial deviation border SD
19	C	Radial gradient border mean
**20**	**F**	**Radial gradient border SD**
**21**	**F**	**Radial deviation border mean (2D)**
22	R	Radial deviation border SD (2D)
23	C	Radial gradient border mean (2D)
24	R	Radial gradient border SD (2D)
25	C	Radial deviation outside mean
26	C	Radial deviation outside SD
27	C	Radial gradient outside mean
28	C	Radial gradient outside SD
**29**	**F**	**Radial deviation outside mean (2D)**
**30**	**F**	**Radial deviation outside SD (2D)**
31	C	Radial gradient outside mean (2D)
**32**	**F**	**Radial gradient outside SD (2D)**
**33**	**F**	**Radial deviation outside-tumor separation mean**
**34**	**F**	**Radial deviation outside-tumor separation SD**
**35**	**F**	**Radial gradient outside-tumor separation mean**
**36**	**F**	**Radial gradient outside-tumor separation SD**
37	R	Radial deviation outside-tumor separation mean (2D)
38	R	Radial deviation outside-tumor separation SD (2D)
39	R	Radial gradient outside-tumor separation mean (2D)
40	C	Radial gradient outside-tumor separation SD (2D)
**41**	**F**	**Radial deviation outside-border separation mean**
**42**	**F**	**Radial deviation outside-border separation SD**
43	C	Radial gradient outside-border separation mean
44	C	Radial gradient outside-border separation SD
45	R	Radial deviation outside-border separation mean (2D)
46	R	Radial deviation outside-border separation SD (2D)
47	C	Radial gradient outside-border separation mean (2D)
**48**	**F**	**Radial gradient outside-border separation SD (2D)**

F = Final set of features that were analyzed after non-reproducible (R) features and correlated features (C) were removed.

Supplementary Table 5: Semantic features analyzed

No.	Features	p-value
1	Fissure attachment	0: absence 1: presence
2	Pleural attachment	0: absence 1: presence
3	Bubble-like lucency / cavitation	0: absence 1: presence
4	Air bronchogram	0: absence 1: presence
5	Calcification	0: absence 1: presence
6	Attachment to vessel	0: absence 1: presence
7	Pleural retraction	0: absence 1: presence
8	Lobulation	0: absence 1: presence
9	Spiculation	0: absence 1: presence
10	Concavity	0: absence 1: presence
11	Attenuation pattern[1]	1: GGO[2] 2: part-solid 3: solid
12	Shape	1: round/oval 2: irregular
13	Border definition	1: well-defined 2: neither 1 or 3 3: poorly defined

[1]None of the tumors were GG0s.
[2]GGO: Ground glass opacity.
